# Preoperative Computed Tomography-Derived Bone Densities in Hounsfield Units at Implant Sites Acquired Primary Stability

**DOI:** 10.5402/2011/678729

**Published:** 2011-11-30

**Authors:** Kyou Hiasa, Yasuhiko Abe, Yohei Okazaki, Keisuke Nogami, Wataru Mizumachi, Yasumasa Akagawa

**Affiliations:** Division of Cervico-Gnathostomatology, Department of Advanced Prosthodontics, Graduate School of Biomedical Sciences, Hiroshima University, Hiroshima 734-8553, Japan

## Abstract

The purpose of this study was to evaluate preoperative CT-derived bone densities in Hounsfield units (HU) at implant sites that acquired primary stability, and to compare these values to the optimal bone densities proposed in the literature. Fifty-one patients, 18 males (37 implant sites) and 33 females (67 implant sites) between 2003 and 2010 were assessed. CT data for different jaw sections, regions, and operating procedures were compared using the Kruskal-Wallis test and Scheffe's test for multiple comparisons (*P* < 0.05). The mean bone density in the maxilla was significantly lower than that in the mandible (*P* < 0.05); the mean bone densities in the 4 jaw regions decreased in the following order: anterior mandible > anterior maxilla > posterior mandible > posterior maxilla. The bone densities assessed by HU fell into the range of optimal bone densities associated with acquired primary implant stability proposed in the literature.

## 1. Introduction

 Oral implant success is mainly influenced by bone quality and bone quantity. Bone quality is an important factor for achieving primary implant stability. Lekholm and Zarb [1] suggested a bone classification system based on macrostructure, where the morphology and distribution of cortical and trabecular bones determine bone quality. However, Lindh et al. [2] proposed a new classification system using reference images to assess the trabecular pattern in periapical radiographs in the mandible before implant treatment, because the accuracy of the classification system of Lekholm and Zarb could not be evaluated. Following the introduction of multislice computed tomography (CT) for preoperative evaluation of bone density in Hounsfield units (HU) as a parameter of bone quality, Norton and Gamble [3] proposed a new classification system based on bone HU and related this new classification to that of Lekholm and Zarb [1]. Recently, the use of cone-beam (CB) CT in dentistry has increased, because CBCT is associated with benefits such as increased patient comfort, lower radiation doses, and lower operation costs compared to conventional CT [4]. However, Nackaerts et al. [5] demonstrated that density profiles of conventional CT showed stable HU values whereas intensity values in CBCT images are not reliable because the values are influenced by the device used, imaging parameters, and positioning. Accordingly, Naitoh et al. [6] found that the trabecular bone volume per total tissue volume obtained using CBCT images was closely correlated with HU values generated from conventional CT images. 

 Primary implant stability has been acknowledged as an essential criterion for later achievement of osseointegration [7]. Primary stability is assessed by resonance frequency analysis using the Osstell device, for which damping capacity assessment is considered reliable, reproducible, and easy to use [8]. Veltri et al. [9] demonstrated that, for optimal implant stability, damping values should be in the range of satisfactory values proposed in the literature, although these values are not related to the radiographic trabecular bone pattern. Lachmann et al. [10] suggested that the Osstell device may be useful for monitoring the state of an individual implant over time, because the outcome of implant stability depends on environmental factors such as bone quality and implant geometry. Merheb et al. [7] showed that a significant linear relationship exists between damping values and HU values at implant insertion and suggested that preoperative evaluation of cortical thickness and trabecular bone HU appears to be the most reliable method for predicting implant stability.

 The purpose of this study was to evaluate preoperative CT-derived bone densities in HU and their association with acquired primary implant stability, and to compare these values to the optimal bone densities proposed in the literature.

## 2. Materials and Methods

### 2.1. Patients and Implants

 Fifty-one patients (18 males, average age 55.8 ± 12.3 years, range 19–76; 33 females, average age 57.1 ± 10.7 years, range 29–77) who had undergone implant placement between 2003 and 2010 using the Strauman Dental Implant System (Basel, Switzerland) or GC Implant Re System Genesio (Tokyo, Japan) were selected for this study (Table 1). None of the patients selected suffered from a systemic disease. Implants were placed via a surgical template for optimal localization and optimal acquired primary stability. This study was approved by the ethics committee of Hiroshima University Hospital (no. 214).

### 2.2. Bone Density

 To evaluate the bone density of each patient before implant planning, a CT scanner (Aquilion TSX-101A; Toshiba Medical Systems, Otawara, Japan) with the following technical parameters was used: 0.5 s, 0.5 mm slice thickness, 0.3 mm slice increment, 135 kV, 150 mA, and 0° gantry angulation. CT images were stored in DICOM format. The images were loaded onto SimPlant software (Materialise Dental Japan, Tokyo, Japan), which enables the construction of a 3 dimensional model of each maxilla or mandible and the determination of bone densities to a thickness of 1 mm inside and 1 mm outside the simulated implant. Bone density was measured in HU. Software-based analysis of bone densities was performed by 1 operator determining the position of implant in the optimum size, type, and angulation. The data were analyzed using the Kruskal-Wallis test and Scheffe's test for multiple comparisons (*P* < 0.05).

## 3. Results

The study cohort included 37 implant sites in male participants and 67 implant sites in female participants; the mean bone density (725.7 ± 174.7 HU) in males was significantly higher than that (546.9 ± 209.7 HU) in females (*P* < 0.01; Table 1). In females, the mean bone density (474.2 ± 230.4 HU) inside the implant was significantly lower than that (619.6 ± 208.8) outside the implant (*P* < 0.01).

 The bone densities in HU for different jaw sections (maxilla and mandible), regions (anterior and posterior), and operating procedures (1- and 2-stage) are shown in Table 2. The mean bone density (540.9 ± 201.9 HU) in the maxilla (31 implant sites) was significantly lower than that (640.0 ± 214.9) in the mandible (73 implant sites; *P* < 0.05). The mean bone density (695.0 ± 206.0 HU) in the anterior region (15 implant sites) was higher than that (596.2 ± 214.3 HU) in the posterior region (89 implant sites), but this difference was not significant (*P* > 0.05). The mean bone density (630.8 ± 223.1 HU) in patients who had undergone the 1-stage procedure (66 implant sites) was higher than that (575.2 ± 198.1 HU) in patients who had undergone the 2-stage procedure (38 implant sites), but this difference was not significant (*P* > 0.05).

 The mean bone density was highest in the anterior mandible (843.6 ± 241.9 HU), followed by the anterior maxilla (640.9 ± 172.7 HU), the posterior mandible (628.2 ± 209.2 HU), and the posterior maxilla (486.0 ± 199.2 HU; Table 3). There were significant differences among 4 jaw regions (*P* < 0.05), but the difference between each region was not significant (*P* > 0.05).

## 4. Discussion

 In this study, which assessed preoperative CT-derived bone densities in HU, the mean bone density in males was significantly higher than that in females, which is similar to findings reported by Turkyilmaz et al. [11]. Likewise, the tendency for the mean bone densities of the 4 jaw regions to decrease, as seen in this study, in the order of anterior mandible, anterior maxilla, posterior mandible, and posterior maxilla is similar to that observed in previous studies [3, 11–15].

A summary of the previously described bone density classification systems [1, 3, 12, 16–18] is shown in Table 3. In the Lekholm and Zarb classification [1], bone density is graded as follows: Q1, almost the entire jaw is comprised of homogenous compact bone; Q2, a thick layer of compact bone surrounds a core of dense trabecular bone; Q3, a thin layer of cortical bone surrounds a core of dense trabecular bone; and Q4, a thin layer of cortical bone surrounds a core of low-density trabecular bone. Bone quality scoring in the rage of Q2 and Q3 is associated with good prospects for implant success, easier implant placement, better primary fixation, and the use of standard instruments and components. Misch [16] defined 4 bone density classes (D1, D2, D3, and D4) based on the clinical drilling resistance of the bone. Based on the Misch classification [16], Trisi and Rao [17] attempted to establish a quantitative threshold of bone volume (%) for the 4 classes and suggested combining D2 and D3 into 1 group, thus classifying bone density into 3 groups of clinical interest: D1, D2/D3, and D4. For bone quality assessment in accordance with the Lekholm and Zarb classification [1], Norton and Gamble [3] found a strong correlation between HU values and sites classified as Q1 and Q4, and thus proposed unifying Q2 and Q3. In addition, they applied the bone density range in HU to the Lekholm and Zarb classification [1]. The CT software used in this study was the same as that used for all previous bone density classifications, and most of the bone densities observed for the different jaw regions in this study were in the range of corresponding optimal bone densities described in these classification systems. Because the bone density range in HU proposed by de Oliveira et al. [12] focused on the trabecular bone only, the bone densities observed in the present study were higher, as the latter included both the cortical and trabecular bone. Recently, Rebaudi et al. [18] introduced a novel bone quality/density classification system that divides bone into 3 classes: hard/dense (H), corresponding to Q1 and D1; normal (N), corresponding to Q2/Q3 and D2/D3; soft (S), corresponding to Q4 and D4 (HNS classification). Hard bone (>1,000 HU) and soft bone (<400 HU) are associated with a higher risk of implant failure whereas normal bone (400–1,000 HU) represents a safe zone. In this study, the optimal bone density range for primary implant stability was set at 400–1,000 HU, corresponding to the classification of normal bone proposed by Rebaudi et al. [18], which indeed seems to lead to implant success. Furthermore, the mean bone density in patients who underwent the 1-stage procedure was justifiably higher than that in patients who underwent the 2-stage procedure, as the former bone densities belonged to the category of normal bone.

 Stoppie et al. [19] demonstrated that predictions of the mechanical properties of the trabecular bone are only valid for implant sites fully situated in the trabecular bone or with a very small amount of cortical bone involvement. This observation indicates that bone densities assessed by HU, as performed in the current study, may be more accurate for the prediction of primary implant stability, except in jaws with a thicker cortical layer. Indeed, Farré-Pagès et al. [20] concluded that HU can be used as a diagnostic parameter to predict possible implant stability. Thus, preoperative assessment of bone densities by HU is very important for optimizing primary implant stability.

## 5. Conclusions

In this study, the mean HU bone density in the 4 jaw regions decreased in the following order: anterior mandible, anterior maxilla, posterior mandible, and posterior maxilla, which is similar to reported reference values. Furthermore, the mean bone densities predicted to maximize acquired primary implant stability, as assessed by HU, were in the optimal range proposed in the literature. CT using HU is therefore a suitable assessment tool for bone densities prior to dental implantation.

## Figures and Tables

**Table 1:** Bone densities in Hounsfield units (HU) for males and females.

			Bone densities (HU)		
Gender	Number of implant sites	Age, years (SD)	Inside (SD)	Outside (SD)	Combination of inside and outside (SD)
Male	37	55.8 (12.3)	700.2 (185.2)^a^	751.1 (236.4)	725.7 (174.7)^c^
Female	67	57.1 (10.7)	474.2 (230.4)^a,b^	619.6 (208.8)^b^	546.9 (209.7)^c^

The presence of the same superscript letter indicates a significant difference (all cases: *P* < 0.01).

**Table 2:** Bone densities in Hounsfield units (HU) for different jaw sections, regions, and operating procedures.

		Bone densities (HU)		
	Number of implant sites	Inside (SD)	Outside (SD)	Combination of inside and outside (SD)
Jaw section				
Maxilla	31	523.8 (267.5)	558.1 (202.2)^a^	540.9 (201.9)^b^
Mandible	73	567.6 (228.7)	712.4 (222.3)^a^	640.0 (214.9)^b^
Region				
Anterior	15	659.6 (270.1)	730.4 (279.1)	695.0 (206.0)
Posterior	89	536.9 (232.1)	655.6 (216.9)	596.2 (214.3)
Procedure				
1-stage	66	571.5 (233.8)	690.1 (235.6)	630.8 (223.1)
2-stage	38	525.1 (251.9)	625.2 (207.6)	575.2 (198.1)

The presence of the same superscript letter indicates a significant difference (^a^ *P* < 0.01; ^b^ *P* < 0.05).

**Table 3:** Bone classifications and bone densities in Hounsfield units (HU) observed in this study and previous studies for different jaw regions.

	Jaw region											
	Anterior mandible			Anterior maxilla			Posterior mandible			Posterior maxilla		
Bone classification												
Lekholm and Zarb [1]	Q1			Q2/Q3						Q4		
Misch [16] Trisi and Rao [17]	D1			D2/D3						D4		
Norton and Gamble [3]	>850 HU			500–850 HU						0–500 HU		
de Oliveira et al. [12]	>400 HU			200–400 HU						<200 HU		
Rebaudi et al. [18]	Hard/dense (H)			Normal (N)						Soft (S)		
	>1,000 HU			400 to 1,000 HU						<400 HU		

Bone densities (HU)	*n*		(SD)	*n*		(SD)	*n*		(SD)	*n*		(SD)

This study	4	844	(242)	11	641	(173)	69	628	(209)	20	486	(199)
Norton and Gamble [3]	25	970	(269)	42	696	(244)	45	670	(249)	27	417	(227)
Turkyilmaz et al. [11]	62	912	(238)	31	751	(181)	37	698	(205)	28	467	(124)
Shapurian et al. [13]	42	559	(208)	45	517	(177)	78	321	(132)	54	333	(199)
Turkyilmaz et al. [14]	58	945	(207)	24	716	(190)	28	674	(227)	21	455	(122)
de Oliveria et al. [12]	6	383	(243)	6	370	(177)	34	306	(187)	29	256	(184)
Fuh et al. [15]	15	530	(161)	47	516	(132)	55	359	(150)	37	332	(136)

Lekholm and Zarb classification based on bone macrostructure: Q1, almost the entire jaw is comprised of homogenous compact bone; Q2, a thick layer of compact bone surrounds a core of dense trabecular bone; Q3, a thin layer of cortical bone surrounds a core of dense trabecular bone; Q4, a thin layer of cortical bone surrounds a core of low-density trabecular bone. Misch classification based on the clinical drilling resistance of the bone: D1, oak or maple wood; D2, white pine of spruce wood; D3, balsa wood; D4, styrofoam. *n*: number of implant sites.
